# Persuasive Systems Design Features of Smartphone Apps for Psychosis: Systematic Review

**DOI:** 10.2196/81101

**Published:** 2026-05-07

**Authors:** Theresa Taylor, Jasmin Peat, David Kell, Shadi Daryan, Pamela Jacobsen

**Affiliations:** 1 Department of Psychology, University of Bath, Claverton Down, Bath, BA2 7AY, United Kingdom, +44 1225 383843

**Keywords:** persuasive systems design, psychosis, schizophrenia, human computer interaction, smartphone app, mhealth, app

## Abstract

**Background:**

It is unclear why some smartphone apps designed for people with psychosis are engaging, while others are not. One possible explanation is the apps’ persuasive features and the operationalization and implementation of these features.

**Objective:**

This systematic review set out to quantify and describe the persuasive features used in smartphone apps for psychosis, investigate whether there was any association between persuasive features and attrition or adherence rates. and document the quality of the included apps.

**Methods:**

We searched electronic databases PsycINFO, PubMed, and Google Scholar for eligible papers published between the years of 2013 and 2025. Hand searches of reference lists were completed. Apps were selected if they were designed for people with psychosis and there were published empirical studies investigating the apps. Two reviewers from the review team (TT, JP, DK, and SD) independently screened papers and extracted data on adherence and attrition, as well as coded papers for evidence of persuasive features according to the persuasive systems design (PSD) model. Available data were synthesized descriptively and narratively. We attempted to access apps via app stores or by correspondence with the research team.

**Results:**

We found 22 apps for psychosis, with 30 associated published papers. The persuasive features were as follows: personalization (18 apps); reminders (15 apps); suggestions (11 apps); tunneling and self-monitoring (10 apps); reduction (9 apps); liking (8 apps); social role (6 apps); rehearsal, praise, and similarity (5 apps); rewards (4 apps); simulation, real-world feel, and social learning (3 apps); surface credibility and normative influence (2 apps); and trustworthiness, social comparison, and social facilitation (1 app). Expertise, authority, third-party endorsements, verifiability, cooperation, competition, and recognition were present in zero apps. Features in the categories of primary task support and dialogue support were well represented, while social support and system credibility support were underused. It was found that there was no association between the number of persuasive features and attrition; an association between persuasive features and adherence could not be assessed. The quality of the apps could not be judged due to 20 of the 22 apps being inaccessible either through the research papers’ authors or through app stores.

**Conclusions:**

Our findings indicate that in psychosis apps there is potential to include a broader range of persuasive features, which might maximize engagement. Psychosis apps may benefit from incorporating more features that leverage the persuasive impact of having users interact (social support) and incorporating features that emphasize system credibility and trustworthiness. Further studies could determine whether an increase in the number of persuasive features will impact app engagement and which features are most impactful in this context.

## Introduction

### Background

There is a well-recognized global gap between the need for and provision of services to prevent, identify, and treat mental health conditions [1]. People who experience psychosis are among those with the highest need for support [2]; thus, interest and research into digital treatments for this condition have grown over the last decade. A recent meta-analysis of randomized controlled trials (RCTs) assessing apps designed for symptom reduction in patients with schizophrenia found statistically significant symptom reduction across positive (standard mean difference [SMD]=−0.205, 95% CI −0.388 to −0.022) and negative symptoms (SMD=−0.406, 95% CI −0.791 to −0.020) [3]. However, adherence to smartphone apps for mental health is notoriously low in both trial and real-world conditions. For example, a systematic review of 93 popular publicly available mental health and well-being apps found that only 3.3% (IQR 6.2%) of users were still accessing the app after 30 days [4]. Specifically relating to mental health apps, a meta-analysis of 70 RCTs found that adherence consistently declined with time [5]. One potential contributor to disengagement is insufficient attention to system design and how it affects users’ experience of apps [6]. Increasing researcher and app designer understanding of the role the technology is playing in digital interventions has the potential to shift patterns of user disengagement [7].

### Prior Work

One area of system design that can be applied to app development is persuasive systems design (PSD). The PSD is defined as the use of computer-mediated products that aim to influence or change people’s cognition and behaviors [8]. A framework has been developed that transforms persuasive principles into software requirements and system features, namely primary task support, dialogue support, system credibility support, and social support [9]. For example, an app praising a user after they complete a session is a system feature of the PSD principle “dialogue support,” which pertains to the feedback an interactive system provides to its users to help them move toward their goal.

It has been hypothesized that persuasive features will increase alliance, adherence, and engagement, in turn leading to better treatment outcomes. Kelders et al [10] conducted a systematic review of web-based health interventions and found persuasive features accounted for a significant difference in adherence scores. However, more recent research on apps has produced mixed results. A review of 70 apps for health and wellness available to the public found a positive correlation between apps’ ranks of popularity (based on ratings and installation) and the apps’ persuasive features [11]. In contrast, Alqahtani et al [12] found no link between the number of persuasive features in mental health apps and efficacy or engagement. Interestingly, a recent meta-analysis looking at efficacy, engagement, and persuasive design in smartphone apps for depression and anxiety found persuasive features were linked to increased effect sizes; however, an increased number of persuasive features was also linked to rising attrition [13]. Thus, the relationship between persuasive design, efficacy, and engagement is multifaceted and opaque. It may be that for some client groups, persuasive design or over-application of persuasive design has the opposite of the intended impact, causing users to disengage. This requires further investigation.

There is evidence that motivation type [12] and psychological and demographic characteristics [14] impact mobile health users’ proclivity to persuasive design, meaning the characteristics of the user may be relevant to understanding how they relate to an app’s features. People with psychosis may have unique needs. For example, a review on the implementation of digital interventions with people with psychosis and bipolar disorder found that this client group may have cognitive impairments that make engagement with complex interventions challenging [15]. A related difficulty for people with serious mental illness is difficulties in social functioning [16,17]. Furthermore, in human-to-human psychosis interventions, the quality of the therapeutic alliance has been found to be associated with treatment outcome; thus, the quality of this client group’s interaction with the therapeutic agent (app) may be paramount to the intervention’s success [18]. Thus, understanding how apps are being designed for people with psychosis and the links between system design and app adherence could provide valuable information for improving these interventions going forward.

### Goal of This Study

There is a gap in the literature for a review that looks in a granular manner at persuasive features within mobile apps designed for people with psychosis. This review aimed to answer the following questions: (1) what PSD features do apps aimed at people with psychosis have? (2) Which PSD features of smartphone apps aimed at people with psychosis are associated with app adherence and attrition rates? (3) What is the quality of apps aimed at people with psychosis?

## Methods

### Overview

This study’s protocol was preregistered on the Open Science Framework [19]. The data associated with this review are also available at the same location on the Open Science Framework. The electronic databases searched were PsycINFO, PubMed, and Google Scholar. Due to the fast-evolving nature of technology, database searches were limited from the year 2013 onward and searched in November 2023 and March 2025, respectively. Query strings were (smartphone* or “mobile phone” or “cell phone”) AND (“app” or “apps” or “application” or “applications”) AND (“schizophrenia” or “schizo” or “psychosis” or “psychotic”). To identify articles not found in the original searches, we reviewed the reference lists of included papers for relevant apps. The authors also used prior knowledge of apps to identify additional inclusions and linked these with their associated papers. Where papers could not be accessed, study authors were contacted.

### Study Eligibility and Screening

The “unit of interest” in this review was the smartphone apps themselves, as we were aware that multiple papers may be published relating to various aspects of the same app (eg, pilot studies, clinical trials, and qualitative papers). We linked together multiple papers where they related to the same app and included those most relevant for gathering the information for this paper.

Inclusion criteria for eligible apps were as follows:

1. The app has associated with it a minimum of one peer-reviewed journal article reporting empirical data.
2. The app is aimed at people with psychosis.
3. The study sample has been confirmed to have psychosis via a prior diagnosis, psychometric measures of symptoms clinically relevant to psychosis, or a clinical interview.
4. App features were sufficiently described in the associated publications, provided by the authors upon request, or the intervention was accessible to the review team.

Exclusion criteria were as follows:

1. The app targets medication adherence, another condition (eg, cannabis addiction, smoking, or diabetes), or is aimed at supplying the user information (eg, on their legal rights).
2. The app is aimed only at monitoring or accessing treatment records.
3. Other digital interventions, for example, computer-based interventions, text, email, online counseling, and videoconferencing.
4. Apps that require other digital devices (eg, virtual reality [VR] headsets).
5. Apps targeting families of people with psychosis or the care team.

Covidence was used for all stages of review, including screening and data extraction. After duplicate records were excluded, 2 reviewers (TT, DK, SD, and JP) independently screened each paper at both title and abstract and full-text review. All screeners completed training, and a consensus check on a small selection of papers was done to ensure consistency between screeners. The initial agreement between screeners was 81% at title and abstract review and 70% at full-text review. Where there were conflicting decisions between screeners, consensus was reached by discussion and with input from the senior author (PJ) when needed.

### Data Extraction

Relevant data from included studies were extracted in duplicate independently by 2 reviewers (TT or JP and DK or SD) using a predetermined data collection tool. Oinas-Kukkonen and Harjumaa [9], which lays out descriptions of persuasive features and examples of how the features can be defined, and their implementation was used as a coding guide for determining the persuasive features. Prior to extraction, reviewers piloted the data extraction tool and discussed discrepancies. During extraction, regular meetings allowed discussion of the coding guide and resolution of discrepancies. The review team extracted information on the apps, including the intervention name, a brief description of the app, treatment target, whether the app was automated (used without a human intervention component alongside) or blended (used with an accompanying human therapist), intended frequency of intervention, and intended duration of intervention. Blended interventions with human support may inherently include persuasive elements outside the app interface, which was out of scope for this review.

From the most recent empirical paper on the app, information describing the study was extracted (study design, location, sample size, participant mean age and diagnoses, attrition during trial, and available adherence data). Missing data were reported in the write-up. “Interrater reliability was assessed across all 22 included apps and 28 PSD features (616 coding decisions). Cohen Kappa was κ=0.95 (98.4% observed agreement).”

### Quality Assessment

The reviewers had planned to assess the quality of the apps using the Mobile Application Rating Scale [20], a quality assessment tool for health apps with published psychometrics [21]. The Apple and Android app stores were searched, and the corresponding authors were contacted to request access to the apps. However, only 2 of the 22 included apps could be accessed; thus, assessing app quality could not be done meaningfully, and this was not completed.

## Results

### Overview

**Figure 1.**
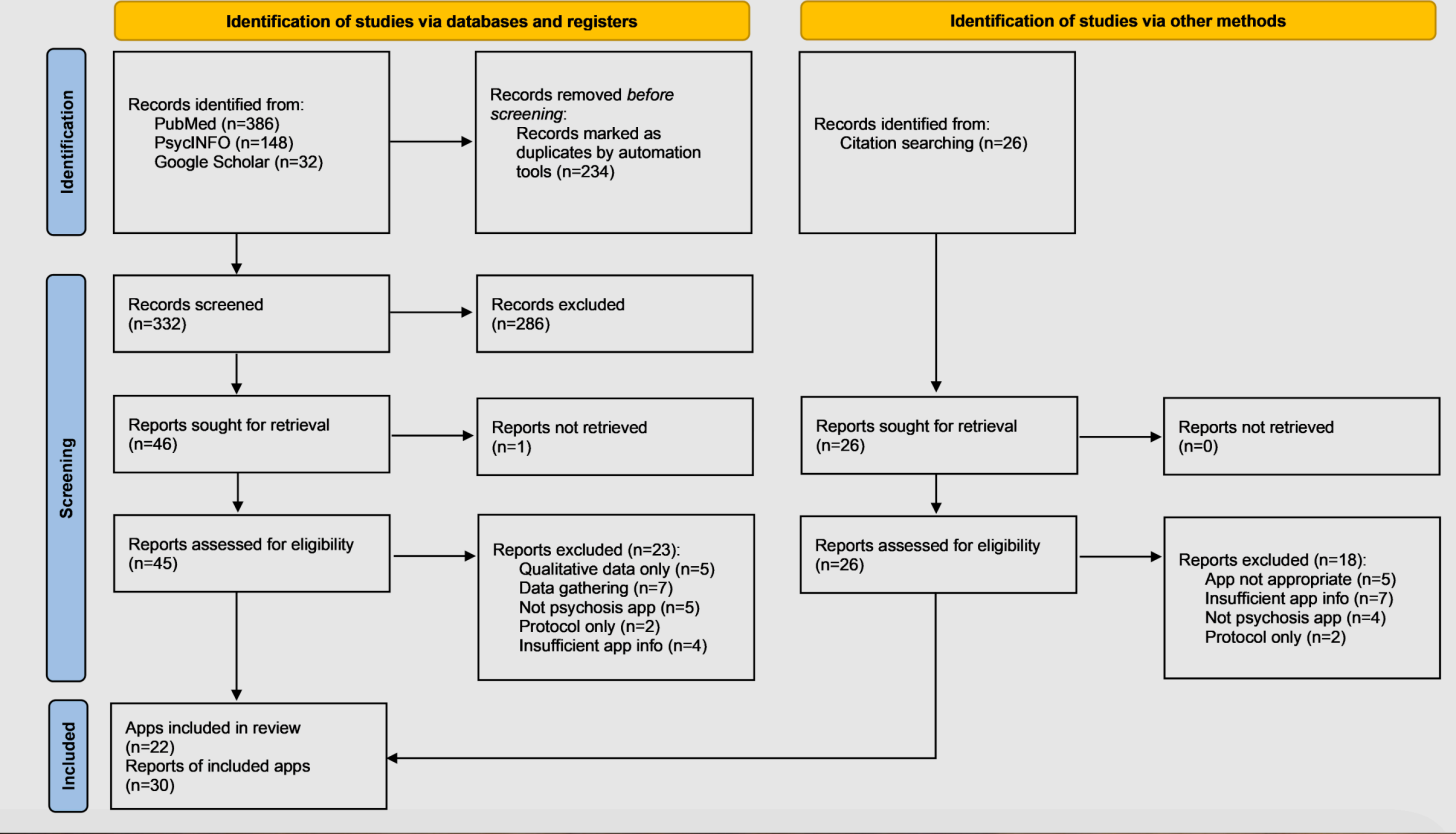
PRISMA (Preferred Reporting Items for Systematic Reviews and Meta-Analyses) diagram of study selection and screening process.

The search identified 566 studies, including 234 duplicates. After title, abstract, and full-text screening, 30 [22-51] eligible papers reporting on 22 apps were included. The screening process is summarized in the PRISMA (Preferred Reporting Items for Systematic Reviews and Meta-Analyses; Checklist 1) diagram (Figure 1). App names, treatment targets, and whether the app is automated or blended are detailed (Table 1). Further information on the apps, associated outcome study characteristics, and attrition and adherence rates is provided (Multimedia Appendix 1) and a matrix of persuasive features of each app (Multimedia Appendix 2). Across 22 apps, 21 categories of persuasive features were identified. Primary task support and dialogue support were the most common, with social support and system credibility support coded considerably less often.

**Table 1.** Table depicting the included app’s names, their treatment target, and whether the apps were an automated or blended intervention.

App name	Treatment target	Automated or blended
A4i (MEMOTEXT Corporation) [22,23]	Medication adherence, personal recovery, and psychiatric symptomatology	Automated
Actissist (University of Manchester) [24]	CBT^a^-informed early psychosis intervention	Automated
ChillTime (Psyxnovation Inc) [25]	Emotional regulation in dual diagnosis psychosis and substance use disorder	Automated
CBT2go (Center for Technology and Behavioral Health; Dartmouth College) [26]	CBT for negative symptoms. Aims to reduce the severity of defeatist performance attitudes	Blended (group therapy+app)
Connect+ (University of Manchester) [27]	Loneliness	Automated
Focus (Northwestern University and University of Illinois at Chicago) [28,29]	Sleep, mood, social, meds, and voices	Automated
Focus-AV (Northwestern University) [30]	Sleep, mood, social functioning, medication adherence, and voices	Automated
Grasp (New York State Psychiatric Institute and Columbia University) [31]	Social cognition and social functioning	Blended (group therapy+app)
IMPACHS (King’s College London) [32,33]	Reducing psychotic and depressive symptom severity	Blended
MASS (University of California Los Angeles) [34]	Social skills training	Automated
Moneo (University of Cambridge) [35,36]	Cognitive training/cognitive rehabilitation	Automated
My Journey (Dartmouth College) [37,38]	Develop self-management skills, achieve self-determined recovery goals, and avoid future relapses	Blended
MCI-S (University Medical Center Hamburg‑Eppendorf) [39]	Metacognitive beliefs, psychotic symptoms, and social functioning	Automated and blended versions
PEAR004 (Pear Therapeutics) [40]	Positive and negative symptoms of schizophrenia	Automated
PRIME (University of California San Francisco) [41]	Motivation, cognition, and negative symptoms	Blended (app+ coaches)
Savvy (University of Groningen) [42,43]	Voice hearing	Blended
Sleep app (University of Oxford) [44]	Sleep	Blended
SlowMo (King’s College London) [45,46]	Paranoia	Blended
SMART app (University of Melbourne) [47]	Improve social functioning and psychiatric symptoms	Automated
TechCare (King’s College London) [48,49]	Positive and negative symptoms of schizophrenia	Automated
TemStem (University Medical Center Utrecht) [50]	Voice hearing, emotionality, and vividness	Automated
WeCOPE (University of Hong Kong) [51]	Improvement in schizophrenia symptoms and personal and social functioning.	Automated

a CBT: cognitive behavioral therapy.

### Research Question 1: PSD Elements

Twenty-one categories of persuasive features were found across the 22 apps [22-51]. The most coded areas of persuasive features were primary task support and dialogue support, with social support and system credibility support being coded significantly less frequently. The maximum number of PSD elements in an app was 11, while the least was 2 (Multimedia Appendix 2).

#### Primary Task Support

The primary task support category includes features that support the user to carry out their primary tasks in the app [9].

#### Personalization

Personalization, defined as the app noting user inputs and adjusting content accordingly, was the most common primary task support feature, present in 18 of 22 reviewed apps [22,24-26,28,30,33,34,37,40-42,44,46,47,49-51]. Personalization encompassed tailored goals [37,41,51], steps toward goals [22,26,34], encouragement [26,44,50] (eg, in the TemStem [University Medical Center Utrecht] app, if the user assessed themselves as feeling “powerless,” an avatar would give them personalized support such as “You are strong!” [50]), using the user’s name [46], customizable skill toolboxes [22,33,40], ecological momentary assessment informing personalized coping strategies [24,28,42,47,49]. The ChillTime (Psyxnovation Inc) app allowed users to give feedback on the personalized coping strategies offered and used artificial intelligence to improve future suggestions [25]. Some apps provided multiple ways to interact with content, such as video or audio [25,30,33], while the WeCOPE (University of Hong Kong) app allowed the user to choose either a male or female voice in audio recordings [51]. Other examples of personalization included customizing the app’s looks [24] and the app triggering a personalized crisis plan [49], and multiple apps allowed users to personalize notification frequency [26,28,37,40]. Hornstein et al [52] suggest 4 personalization levels (intervention content, content order, and guidance/communication level) and 4 underlying mechanisms (user choice, provider choice, decision rules, and machine learning). Most reviewed apps are personalized at the intervention content level, using user choice or decision rules.

#### Tunneling

Tunneling was found in 10 reviewed apps [22,24,26,27,31,33,34,39,41,50]. Tunneling means the app content changes progressively with use, providing various opportunities for the user to be persuaded to make behavioral changes. Observed tunneling strategies included content that changed in a stepwise fashion toward goals [22,26,34,39,41] and progressively more challenging modules [27,41,50].

#### Self-Monitoring

Self-monitoring, a feature enabling users to track symptoms or goal progress, was found in 10 apps [22,24,26,27,33,34,37,41,42,50]. Capturing data and feeding it back to users can encourage behavior change. Several apps provided graphs for self-monitoring [22,24,33,37,41,50], and the IMPACHS app linked self-monitoring with customizable action plans [33].

#### Reduction

Reduction, which simplifies complex behavior into simple tasks, was found in 9 apps [25,27,30,31,33,34,44,50,51]. This feature was implemented through simplified language [25,33], standardized content for familiarity [25], breaking tasks into components [31,33,34], and ensuring tasks were quick to complete [25,27,44].

#### Simulation

Simulation, a feature allowing users to observe cause-and-effect relationships (eg, increased physical activity and weight loss), was found in 3 apps. In all instances, an actor demonstrated a skill and its contribution to achieving the user’s goal [27,30,34].

#### Rehearsal

Rehearsal provides a way for users to rehearse a behavior in the app that they then use in daily life and was present in 6 apps [26,28,30,31,42,46].

#### Tailoring

Tailoring means there are different user groups that are shown different versions of the app, which was featured in 2 apps [34,44].

#### Dialogue Support

Dialogue support incorporates the features a system uses to increase interactivity between the app and the user.

#### Reminders

Reminders are notifications that remind the user of their target behavior, and these reminders were the most widely used dialogue support feature (present in 15 of 22 apps) [22,24-27,31,34,36,37,40,42,44,46,47,49]. Besides encouraging app access, reminders were sent to promote medication adherence [22,36,37,47], symptom assessment [47,49], health care appointments [22], daily activities like hygiene [47], goal-focused behavior change [26,37,44], and coping strategies such as positive self-talk [42]. While the SlowMo (King’s College London) app made notifications optional [46], most apps sent multiple daily notifications without specifying if users could disable them.

#### Suggestion

Suggestion, a feature where apps recommend skills or behaviors to users, was found in 11 apps [22,24-28,33,34,40,41,47]. Most apps stated that various cognitive and behavioral strategies were encouraged without detailing these [22,24,28,33,40,41]. However, some apps clearly defined their suggestions, such as CBT2Go’s (Dartmouth College) behavioral experiments to test beliefs (eg, “Try asking someone to go for a short walk”) [26] and MASS’s (University of California Los Angeles) step-by-step actions toward goals like making a new friend [34]. Apps also varied in applying suggestions; for example, ChillTime suggests random strategies to encourage trying new things [25], while the SMART (University of Melbourne) app matches suggestions to the user’s mood [47].

#### Liking

Liking, the app’s visual appeal to the user, was considered and implemented in 8 reviewed apps [24,25,27,28,33,37,41,46]. Strategies for creating visually attractive apps included using multiple images [24], visual simplicity [25], color [37], user customization of visuals [24,46], and familiar visual formats, such as Connect+’s (University of Manchester) Instagram (Meta Inc)-like design [27].

#### Social Role

The system adopted a social role in 6 reviewed apps [22,26,33,36,37,41], primarily related to user interactions with health care professionals. This included connecting users to health care providers [22], collecting information to facilitate deeper conversations between users and health care professionals [26,33,36,37], and arranging appointments with health care providers [36]. Only the PRIME (University of California, San Francisco) app actively encouraged user-to-user interaction [41] by generating daily prompts like “Share a silly selfie!” or “Write down a list of things you are grateful for” to encourage participants to share responses.

#### Similarity

Similarity, in which the system meaningfully reminds users of themselves (eg, by describing relatable scenarios or using familiar language), was reported in 5 apps [24,27,34,39,44]. Some apps used videos depicting others in comparable situations progressing toward their goals [24,27,34], such as Actisist’s (University of Manchester) video story “William walks away from weed” about a person with psychosis quitting cannabis [24]. Connect+ aimed to imitate youth language for increased relatability [27]. Several apps valued input from people with lived experience to create these features [24,27,34,44].

#### Praise

Praise, a form of positive persuasive system feedback, was found in 5 reviewed apps [26,34,41,44,50]. The MASS app used praise to increase anticipatory pleasure for future social experiences in accordance with the temporal experience of pleasure model in schizophrenia [34]. CBT2Go praised high confidence ratings for goal-related tasks while challenging low ratings [26].

#### Rewards

Rewards are a tactic to reinforce target behaviors and were present in 4 apps [26,27,44,50]. For most apps, rewards were given in the form of points, stickers, or stars; however, the CBT2Go app allowed a selfie to be taken after an achievement [26].

#### System Credibility Support

System credibility support, an umbrella term for the ways a system promotes the perception of its own credibility and therefore increases its persuasiveness, was poorly represented in the reviewed articles. Assessing apps in this area was challenging, as most publications lacked details, and only 2 apps, Actisist and ChillTime [24,25], out of 22 apps, were directly accessible. These 2 apps met the surface credibility threshold (competent look and feel). A real-world feel, where a system provides information about its content creators to build credibility, was found in 3 apps [24,25,30]. Two apps provided details of involved clinicians, such as names and work addresses [24,25], while 2 showed videos of clinicians demonstrating skills or explaining content rationale [24,30]. One app’s qualitative data showed evidence of trustworthiness, with users calling it “honest” [27]. No evidence could be found of third-party endorsements (the system provides endorsements from other sources), authority (the system refers to people in the role of authority), expertise (provides information showing knowledge, experience, and competence), or verifiability (the system provides means to verify the accuracy of the program via outside sources) in the apps or their associated papers.

#### Social Support

Social support encompasses how a system harnesses social influence to motivate users, such as enabling users to observe others’ behaviors or compare their own behaviors to others. By leveraging the persuasive power of social interaction, a system can motivate individuals to achieve their target behavior. Social support was detailed in 4 apps [27,34,39,41]. Social learning, where users observe others performing target tasks or behaviors through the system, was present in 3 apps [27,34,39]. One app used social facilitation, allowing users to discern that others are performing the behavior alongside them, while 2 engaged normative influence, using peer pressure to encourage target behaviors [22,41]. A4i (MEMOTEXT Corporation) included a moderated, anonymized platform for users to share strategies and tips [22]. PRIME used social comparison, facilitating performance comparisons between users, and automatically generated accomplishment posts for completed challenges, providing opportunities for social reinforcement [41]. No examples of competition, cooperation, or recognition were found in the examined apps.

### Research Question 2: Which PSD Features of Smartphone Apps Aimed at People With Psychosis Are Associated With App Adherence and Attrition Rates?

The number of persuasive features and attrition percentage for each app showed no evidence of an association (Pearson *r*=0.07, 95% CI −0.4 to 0.5). However, this finding assumes equal weight for each PSD feature, which is likely unjustifiable. Certain features may be more strongly associated with attrition than others, if any relationship exists, but these data are insufficient for this level of analysis. Inconsistent adherence reporting across apps prevented comparisons regarding adherence-persuasive feature links.

### Research Question 3: What Is the Quality of Apps Aimed at People With Psychosis?

App stores were searched, and corresponding authors were contacted to access all apps. However, only 2 apps were accessible, making a quality assessment impossible, as it would have required opening and using the apps to evaluate each criteria point.

## Discussion

### Principal Results

This review assessed PSD features in mobile apps for people with psychosis, aiming to describe the features, links to adherence and attrition, and app quality. The categories of primary task and dialogue support were well-represented, while social support and system credibility support were underused, potentially impacting app efficacy and engagement, as both social connectivity (social support) and a user having ways to judge a system’s credibility (system credibility support) could impact motivation with this client group. No link was found between persuasive features and attrition; the relationship with adherence and app quality couldn’t be assessed.

### Comparison With Prior Work

No previous study has systematically documented the PSD features present in apps for psychosis. The finding that personalization, self-monitoring, and reminders were among the most common strategies is consistent with previous findings on physical activity, mental health, and health apps [11,12,53]. However, the existence of these features does not guarantee a positive impact on users; how these features are applied is crucial to their utility. For example, reminders were a prominent persuasive feature in the psychosis apps reviewed; however, most apps did not give users a choice in notification frequency. One of the postulates of persuasive systems is that the system should aim to be unobtrusive, and inappropriate or ill-timed reminders may have a reverse of the desired impact [54]. Persuasion depends on symbolic strategies to trigger emotions [55], and a blunt, unskilled approach such as sending countless reminders to use the app per day can provoke a negative emotional response, which is counterproductive. Similarly, self-monitoring is considered a fundamental feature of several evidence-based psychological therapies, including cognitive behavior therapy [56], dialectical behavior therapy [57], mindfulness [58], emotion-focused therapy [59], and acceptance and commitment therapy [60]. Thus, it is logical that psychosis apps would utilize self-monitoring to help users track their feelings, thoughts, and behaviors. However, the apps reviewed in this study limited data gathering to manual input, prompted by several daily notifications. Manual entry of personal data can be time-consuming and tedious [61] and may be a barrier to people with serious mental health difficulties. Thus, in the context of psychosis, it is unclear if self-monitoring promoted engagement in the manner intended or had a reverse to desired impact due to the frequency and high cost to the user of multiple manual inputs per day.

Research indicates that psychological and demographic variables influence susceptibility to persuasion [14]; thus, in planning these interventions, these variables should be considered. For example, in this review, reduction (simplifying tasks) and suggestion (suggesting actions to users) were among the top strategies used. However, in a review of mental health apps, these 2 strategies were used in fewer than 5 of 103 apps [12]. Thus, these strategies, which facilitate ease of app use, may be more widely implemented and necessary in a psychosis sample due to cognitive impairment and disability associated with this client group [15-17].

This study also found that apps relied heavily on the primary task support and dialogue support categories of persuasive design, with social support and system credibility support underrepresented. This means that attempts to be persuasive are unbalanced; there is emphasis on helping users to complete tasks and the interactiveness of the app, while techniques to make the app more trustworthy and credible to users and methods to leverage the impact of social forces to support behavior change are ignored. Social support strategies have been found to be more widely implemented in physical than in mental health apps [62]. However, social support is an important strategy for users who experience mental health issues because it is common to feel isolated or stigmatized, and digital interventions could benefit from intervening at the level of the social rather than limiting treatment targets to internal drivers for poor mental health. Lack of system credibility support is common across multiple types of apps [12,62]. A postulate of persuasive systems is that they should always be open to the user and reveal designer bias behind the system both to protect fairness and increase persuasiveness. We argue that system credibility support is fundamental in this time, where technology is developing at a rapid rate and users cannot be expected to understand a system’s functions without guidance. Users need to be assured of not only the effectiveness, reliability, and evidence base of the app’s content, but also that their data will be protected (privacy). Furthermore, without this in place, it is difficult to advance other features such as passive monitoring if users are not fully assured of the app’s credibility.

Our ﬁndings suggest that the number of persuasive features used in psychosis apps is not linked to attrition rates. However, this is not necessarily an indicator that the persuasive features were not assisting the user to achieve their purpose with the app. Some authors propose that disengagement is not a barrier to efficacy if a user has achieved their goals with the digital intervention at the point of disengagement [63]. For example, a recent meta-analysis looking at efficacy, engagement, and persuasive design in smartphone applications for depression and anxiety found increased persuasive features were linked to increased effect sizes and increased attrition rates [13]. However, there may be other factors explaining the disconnect between increased persuasive features and engagement in this review; for example, there is a lack of evidence on which persuasive features are the most effective at increasing adherence and reducing dropout. Therefore, it is likely that totaling persuasive features is insufficient. Using one appropriate feature is preferable to multiple inappropriate features, too many features from one persuasive category, or a combination of appropriate and inappropriate features [64]. Furthermore, although PSD is commonly used to inform app development, the model does not contain a guideline on operationalizing the features [9]. Consequently, the same persuasive feature can be shaped differently in different interventions. We found some apps with highly creative implementation of PSD, while others executed persuasive features in a minimal manner. This mirrors the results of Sporrel et al [65], who examined 29 physical activity apps and found heterogeneity in how the features were executed, which appeared to influence the effectiveness of the intervention. These diverse implementations mean that interventions with the same persuasive feature might evoke different user responses, rendering it difficult to draw a conclusion about the impact of a specific persuasive feature at the theoretical level. Furthermore, technological advancements mean that some apps used features that did not fit within the PSD framework [53]. Thus, the lack of clarity on how many persuasive features are optimal, the lack of guidance on the operationalization of features, and the lack of acknowledgment of emerging features highlight the need to extend the PSD model. Furthermore, extension of the PSD model could include standardized checklists or algorithms for the implementation of persuasive features as guidance to app developers and reviewers.

This study’s findings provide possible guidance to app developers. The finding that, although reminders are a prominent feature, little choice of their frequency was offered indicates that app engagement may benefit from offering users choice when providing notifications. This review found that self-monitoring was a common feature; however, given the functional impairments this patient group frequently faces, passive monitoring may lower the burden on the user. To implement these more advanced features, user trust is crucial. Thus, given this review’s finding that system credibility features were not common, developers could benefit from including trust-building features such as highlighting the team, evidence, endorsements, backend and data storage of the app system, and real-world institutions behind the app product. This client group faces social isolation, and this review found there was little focus on social support features; thus, developers can consider integrating peer-to-peer interactions or content that gives an indirect indication of peers and others using the app (eg, leaderboards) into apps for psychosis.

### Limitations

This review has several methodological strengths, including a preregistered protocol, a systematic search strategy, and 2 independent raters throughout study selection and data extraction. However, several limitations should be noted.

The most significant constraint on interpretation is that only 2 of the 22 apps could be tested directly, rather than through reading their described features in their associated research papers [24,25]. This means the review may not fully capture the persuasive features present in each app, particularly those that are less tangible or harder to articulate—such as system credibility support—which researchers may be less likely to describe explicitly. The reported prevalence of feature categories should therefore be interpreted with this in mind, as it may reflect limitations in how apps are described in the literature as much as the features themselves.

Identifying persuasive features is also an inherently interpretive process; review teams may conceptualize and operationalize features differently. Relatedly, this review’s feature-counting approach assumes equivalent value across different persuasive features, which may not hold in practice; however, a more nuanced weighting of features was beyond the scope of this analysis.

The absence of a formal risk of bias assessment is a further limitation. Although a quality appraisal of the apps was planned, it was not feasible given their inaccessibility. Additionally, attrition and adherence data were either unreported or reported inconsistently across studies, preventing any meaningful analysis of the relationship between persuasive features and these outcomes. Together, these constraints limit conclusions about feature effectiveness, and further empirical work is needed.

Finally, while non-English articles were not explicitly excluded, studies would only have been identified if their abstracts were available in English, meaning relevant research published in other languages may have been missed.

### Conclusions

Our findings indicate that in psychosis apps there is an uneven use of persuasive features, with most apps relying on primary task support and dialogue support features, while social support and system credibility support remain underrepresented. This may be impacting the efficacy and engagement of these interventions. Psychosis apps may benefit from incorporating more features that leverage the persuasive impact of having users interact (social support). There is a human instinct to respond to social forces, and thus incorporating these domains into psychosis apps may allow developers to engage nonengagers. Incorporating features that emphasize system credibility and trustworthiness to the user is also crucial. As technology advances, there is an increasing need for designer openness about their positioning, bias, and how data are stored and used, and further technological advances can only be widely applied if users have confidence in the systems that gather their data. Future research could explore expanding the PSD model, as this review indicates it no longer encompasses all features apps are using, and although the underlying principles may remain sound, the way the model quantifies and describes features is becoming outdated with rapid technological advancements. Furthermore, there is a lack of clarity on how many persuasive features are optimal, how to operationalize features, and how persuasive categories work together. It may be that certain features are more effective in this context than others, and emphasis should not be on increasing numbers of persuasive features but on those most likely to influence adherence, attrition, and efficacy. In future research, a positive deviance approach could be used to examine the features of the most engaging and effective apps, while correlational or experimental research designs could explore the impact of different features on adherence and attrition. Qualitative interviews with users may shed light on what features are experienced as most impactful by users and contribute to understanding their emotional valence and possible impacts on effectiveness.

## Supplementary material

10.2196/81101 Multimedia Appendix 1 Characteristics of apps included in the review.

10.2196/81101 Multimedia Appendix 2 Table of persuasive systems design (PSD) features present in each app.

10.2196/81101 Checklist 1 PRISMA checklist.

## Abbreviations

PRISMA: Preferred Reporting Items for Systematic Reviews and Meta-Analyses
PSD: persuasive systems design
RCT: randomized controlled trial
SMD: standard mean difference
