# Supplementary material for: Persuasive Systems Design Features of Smartphone Apps for Psychosis: Systematic Review
Source: JMIR Hum Factors. 2026 May 7;13:e81101. doi: 10.2196/81101 (PMC13151958; doi:10.2196/81101)
Supplement: Multimedia Appendix 1 [file humanfactors-v13-e81101-s001.docx]

| **App name** | **Study design** | **Location** | **Sample size, Mean age, Participant diagnoses** | **Brief description of the app** | **Treatment target** | **Automated or blended** | **Intended frequency/duration of use** | **Attrition data)** | **Adherence data** |
| --- | --- | --- | --- | --- | --- | --- | --- | --- | --- |
| A4i [22,23] | Pre post-test design | Canada | 38 participants, mean age 31.4 (SD 8.6), diagnosed with: schizophrenia diagnosis, schizoaffective, psychosis NOS, psychosis comorbid with bipolar, or autism with prominent psychosis symptomatology | The A4i app provides seven functionalities including daily activities, passive data collection, peer support, and self-management such as coping tips. A unique feature is an ambient sound tester to allow users to discriminate ambient sound from auditory hallucinations. | Medication adherence, personal recovery, and psychiatric symptomatology | Automated | One month, twice daily notifications | 0% | The mean number of interactions per day was 4.21 (SD = 5.19), used for a mean of 25.12 days (SD = 6.32; range: 16–46 days) |
| Actissist [24] | Single blind RCT | United Kingdom | 36 participants (24 participants used Actissist +TAU  and 12 participants used ClinTouch + TAU), mean age of 20.21 years (SD = 7.37)—Actissist; mean age of 18.33 years (SD = 7)—ClinTouch, diagnosed with psychosis | App features include non-directive content, recovery videos, calming exercises, psychoeducation, and goal-setting. | CBT informed early psychosis intervention | Automated | 12 weeks, 3 notifications a day, 6 days a week | 21% (Actissist app users) | 75% participants used Actissist at least once/day |
| Chilltime [25] | Pilot (Feasibility + acceptability) | Canada | 13 participants, mean age 27.6 (SD 4.5)**,** diagnosed with schizophrenia spectrum disorder with a comorbid substance use disorder | A total of 20 coping strategies regrouped in four categories (behavioural, emotional, cognitive, spiritual) were included in the app. | Emotional regulation in dual diagnosis psychosis and substance use disorder | Automated | 30 days, notifications twice daily | 18% | Approximately half of the patients (5/11) used the tool at least 33% of the days (11-21 days) while the other half used it 27% of the days or less (5-8 days) during the project. |
| CBT2go [26] | Single-arm, open-trial, pre-post evaluation | USA | 31 participants, mean age 48.3 (SD 9.5) years, diagnosed with schizophrenia or schizoaffective disorder | App features included recovery goal setting, thought challenging, scheduling of pleasurable activities and social interactions and activity tracking. | CBT for negative symptoms. Aims to reduce severity of defeatist performance attitudes | Blended - weekly in-person group therapy with a smartphone app | 24 weeks, daily notifications | 19% | There was a mean of 32.3 (SD 31.5) responses to 168 action plan prompts (19.2%) at 24 weeks |
| Connect+ [27] | Pilot, pre post design | Australia | 12 participants, mean age 20.50 years (SD =2.65), diagnoses of schizophrenia, schizoaffective, schizophreniform and psychotic disorder NOS | The +Connect app aims to reduce loneliness in young adults with  early psychosis by providing exercises to practice positive  interpersonal skills and strengthen relationships. | Loneliness | Automated | 6 weeks, daily notifications | 17% | All but two participants completed the +Connect program, completing 95% (40.10 out of 42 days) of the program. |
| Focus [28,29] | Large, prospective, multicentre longitudinal study | USA | 347 participants. Participants aged <35 years = 199 (57%), aged 35-45 years = 80 (23%), >45 years = 68 (20%). Diagnosed with schizophrenia spectrum disorder and within 60 days of discharge from a psychiatric hospital. | The app provided patients with access to illness management strategies in the areas of medication, mood, social, sleep and voices which they could self-initiate use of. | Sleep, mood, social, meds and voices | Automated | 6 months, notifications up to 3 times a day, *Analysis restricted to on demand access by participants | 6% | There were a total of 75,447 FOCUS logins; 35,739 (47.4%) were self-initiated and 38,139 (50.6%) were off-hours. 18,450 of the logins during off-hours were self-initiated (24.5%). |
| Focus-AV [30] | Mixed methods | USA | 10 participants, mean age of 45.5 years (SD 13.18), schizophrenia or schizoaffective disorder | FOCUS–AV is an adapted version of the FOCUS smartphone intervention with video adaptations for all the FOCUS content | Sleep, mood, social functioning, medications adherence and voices | Automated | One month, notifications up to 3 times a day | 10% | Participants responded to 67% of prompts. On average, participants used interventions 6 days a week, 4 times daily. |
| Grasp [31] | Pilot (Feasibility + acceptability) | United Kingdom | 14 participants, mean age 45.1 (9.78), diagnosed with psychosis‐ spectrum difficulties, | Three types of tasks delivered by the app: stories (short vignettes of social situations), emotions (aimed to target perception) and facts and guesses (aimed to address difficulties with jumping to conclusions in social situations) | Social cognition and social functioning | Group therapy + app | 21 days, 2 notifications per day | 21% | Average number of tasks completed was 70%. Mean number of days the app was used was 12.91 (SD = 7.40). |
| IMPACHS [32,33] | Non-controlled trial | Germany and Denmark | 24 participants, mean age 28.21 years, 75% with a diagnosis of schizophrenia | The CBT-p based smartphone-app includes a psychoeducational manual, eight interactive e-learning-modules on difficulties associated with psychosis, individualizable action plans, a trigger response plan, and self-assessment. | Reducing psychotic and depressive symptom severity | Blended | 6 months, client initiated | 29% | Participants used the solution on M = 2.91 days/week, with more frequent usage in month one (M = 4.25 days/week) than in later months (e.g., month five: M = 2.10 days/week). |
| MASS [34] | Pre post test | USA | 37 participants, mean age 46 (SD 11), diagnosis of schizophrenia or schizoaffective | The MASS app seeks to address social impairment by offering social skills training and administering EMA surveys. | Social skills training | Automated | 60 days, Notifications twice daily, also client initiated | 16% | Participants responded to 42.5% (51/120) of notifications on average. |
| Moneo [35,36] | 1-year multicentre, open-label randomized study | Poland | 199 in intervention, 91 in control, Mean age 32.1 years, diagnosed with paranoid schizophrenia | Cognitive training focusing on response time, correct answer rate, incorrect answer rate, and fatigability to check. Exercises were based on Cognitive Remediation Therapy and each training included three series of exercises, with increasing levels of difficulty. | Cognitive training/cognitive rehabilitation | Automated | 12 months, twice weekly | 29% (intervention) 34% (control) | Fifty participants completed at least one cognitive training per month in the last 4 months of the study. On average, each patient completed 23 training modules. |
| My Journey [37,38] | Two-arm unblinded feasibility RCT. | UK | 40 participants (20 in each arm), mean age 29.7 years (SD 9.78), diagnosed with schizophrenia, schizotypal or delusional disorder | The My Journey 3 app aims to support psychosis patients by providing self-management interventions. The features include relapse prevention plan, symptom and medication tracker, and psychoeducation | Develop self-management skills, achieve self-determined recovery goals and avoid future relapses | Blended | 12 months, user initiated | 17% drop out at 4 months and 25% drop out at 12 months | The median number of My Journey 3 uses was 16.5 (IQR 8.5 to 23) and median total minutes spent using My Journey 3 was 26.8 (IQR 18.3 to 57.3). |
| MCI-S [39] | Quasi-experimental design with a non-equivalent comparison group | Not reported | 50 participants, ages 18-65, diagnosed with schizophrenia | An app based metacognitive intervention program with weekly mentoring sessions. Sessions focused on metacognition, understanding of perspectives, identifying the social context and daily practice. | Metacognitive beliefs, psychotic symptoms, and social functioning | Automated and blended versions | Ten 90-minute app sessions | 8% (app only) 17% (app + mentoring) | No information on adherence |
| PEAR004 [40] | Randomised Controlled Trial | United States | 56 PEAR participants, 56 control, PEAR mean age 43.7 (10.99) control mean age 45.7 (11.60), diagnosed with schizophrenia | PEAR-004 is a self-management app that provides skills, surveys and modules. | Positive and negative symptoms of schizophrenia | Automated | 12 weeks, 3 notifications per day | 14% drop out of PEAR sample | 4.1 (SD 1.7) sessions/day, 4.2 (SD 3.4) hours/day |
| PRIME [41] | Double blind RCT | 27 US states and 7 countries (Canada, Mexico, UK, France, Australia, India, Israel) | 100 participants (48 in PRIME and 52 in control), mean age PRIME = 33.98 (10.6) and control 33.58 (10.89), Psychosis spectrum disorder (schizophrenia, schizoaffective, schizophreniform, psychosis (NOS), bipolar, MDD with psychotic features | PRIME techniques include CBT, behavioural activation, mindfulness, psychoeducational approaches, and coaching. PRIME participants  message and interact with their coach as well as a community  of peers. | Motivation, cognition, and negative symptoms | Blended (app + computer + coaches) | 16 weeks, 2 hours per day | 22% | Participants in the PRIME group averaged 17.15 (SD 13.89) hours of cognitive training across the study, with an intensity of 1.33 (SD 0.98) hours per week. |
| Savvy [42,43] | single-blind, parallel group, pilot RCT | Australia | 34 participants (17 intervention 17 control), mean age SAVVY 39.12 (10.64) mean age control 42.59 (10.64), bipolar w. psychotic features, major depression w. psychotic features, schizoaffective disorder, schizophrenia, schizophreniform, unspecified schizophrenia spectrum disorder | The app content is composed of two phases: identifying and implementing individualised coping strategies which then are supported by personalised EMI reminders in daily life. | Voice hearing | blended | 4 weeks - EMI notifications 5x per day | 24 | The average completion rate of the daytime EMA questionnaires was 72%. Completion rates of evening EMA questionnaires across the intervention was 74%. Scheduled EMI reminders were viewed on average 2.5 times per day, and 1.5 times per day when user-initiated |
| Sleep app [44] | Single-arm, uncontrolled study | United Kingdom | 14 participants, mean age 35.57 (SD 10.88), diagnosis of first episode psychosis, schizophrenia, schizoaffective disorder | The smartphone app intervention draws on CBTi techniques, adapted for individuals with psychosis. Six core weekly modules, and one further participant-chosen module (‘Managing Worry’ or ‘Coping with Voices’). | Sleep (CBTi) | Blended | 6 weeks | 21.40% | The mean number of modules engaged with was 5.6 (SD = 1.8). On average, participants engaged with 13.7 (34.3%; SD = 10.4) of the 40 reminders. |
| SlowMo [45,46] | Parallel-arm, assessor-blinded, randomized clinical trial | UK | 361 participants, 181 randomised to SlowMo, 180 to TAU, mean age 42.6 (11.6),diagnosed with schizophrenia, psychosis (other), delusional disorder, schizoaffective disorder | SlowMo is a digitally supported CBTp consisting of 8 individual, face-to-face sessions (60-90 minutes) in accordance with a clinical manual that was delivered within 12weeks. The web app synchronises to a native android mobile app providing access in daily life to SlowMo strategies and individualized safer-thought bubbles | Paranoia | Blended | 12 weeks, app used on demand | 8% | Mobile app adherence was operationalized as at least 1 home screen interaction after a minimum of 3 therapy sessions. Therapy fidelity was high; of the 168 individuals who attended at least 1 session, 159 (94.6%) met a priori criteria for web app delivery, and 100 of 140 (71.4%) met adherence criteria for mobile app use |
| SMART app [47] | Pre and posttest, feasibility and effectiveness | Netherlands | 64 participants (27 participants receiving ESM-derived personalized feedback and 23 participants without feedback), mean age of 37.9 years (SD = 8.6)— feedback; mean age of 40.3 years (SD = 10.9)—no feedback, diagnoses of schizophrenia, schizopaffective disorder, psychotic disorder, schizophreniform disorder | ESM app with personalised activity suggestions based on ESM. Feedback was provided on psychotic symptoms, social engagement, health behaviour, physical activity, and mood and emotion. | Improve social functioning and psychiatric symptoms | Automated | 21 days, 6 ESM notifications daily, 2 personalised prompts daily | 22% | The response rate was 64% for the ESM questionnaires. In the feedback group, participants indicated that on 49% of the ESM days they acted on at least one personalised feedback prompt per day. |
| TechCare [48,49] | Mixed methods feasibility study | UK | 16 participants (4 in test-run study, 12 in feasibility study), mean age of 24.83 years (SD = 4.83), Participants recruited from Early Intervention Services (for psychosis) | The TechCare app aims to reduce relapse by improving user coping strategies and monitoring user symptoms and responses. | Positive and negative symptoms of schizophrenia | Automated | 6 weeks, 3 notifications per day | 17% drop out (for feasibility sample) | 66.67% of participants engaged with ≥33% of the app’s notifications. Participants used the app on average 1.88 times per day. |
| TemStem [50] | Naturalistic study, pre and post tests | Netherlands | 1048 participants, mean age 35.34 (SD 14.03), self identifyying as voice hearing | After psychoeducation about voice hearing, Temstem offers two functions: one designed to inhibit voice activity through the processing of incompatible language and one which uses tasking to decrease memory vividness and emotionality | Voice hearing, emotionality, and vividness | Automated | Data was collected over a two and a half year period between 2017 and 2019. App use user initiated. | No drop out due to being a naturalistic study | The total number of Temstem sessions per user ranged from 2 to 110 (total 16,235 games, M = 15.49, SD = 16.88). |
| WeCOPE [51] | Pretest and posttest design | Portugal | 9 participants, mean age of 38 years (SD±9.701), diagnosis of schizophrenia | The WeCOPE app has four modules: symptom monitoring, problem solving, anxiety management and goal setting. | Improvement in schizophrenia symptoms and personal and social functioning. | Automated | 8 weeks, user initated app use. | Unclear in study reporting | The majority of participants (45%) used the app two or three times a week. |

References:

22. Kidd SA, Feldcamp L, Adler A, et al. Feasibility and outcomes of a multi-function mobile health approach for the schizophrenia spectrum: App4Independence (A4i). PLOS ONE. 2019;14(7):e0219491. [doi: ] [Medline: 31306439]

23. Kidd SA, D’Arcey J, Tackaberry-Giddens L, et al. App for independence: A feasibility randomized controlled trial of a digital health tool for schizophrenia spectrum disorders. Schizophr Res. Jan 2025;275:52-61. [doi: ] [Medline: 39657429]

24. Bucci S, Barrowclough C, Ainsworth J, et al. Actissist: Proof-of-Concept Trial of a Theory-Driven Digital Intervention for Psychosis. Schizophr Bull. Aug 20, 2018;44(5):1070-1080. [doi: ] [Medline: 29566206]

25. Pennou A, Lecomte T, Potvin S, et al. A Mobile Health App (ChillTime) Promoting Emotion Regulation in Dual Disorders: Acceptability and Feasibility Pilot Study. JMIR Form Res. Jan 27, 2023;7(1):e37293. [doi: ] [Medline: 36705963]

26. Granholm E, Holden J, Dwyer K, Mikhael T, Link P, Depp C. Mobile-Assisted Cognitive Behavioral Therapy for Negative Symptoms: Open Single-Arm Trial With Schizophrenia Patients. JMIR Ment Health. Dec 1, 2020;7(12):e24406. [doi: ] [Medline: 33258792]

27. Lim MH, Gleeson JFM, Rodebaugh TL, et al. A pilot digital intervention targeting loneliness in young people with psychosis. Soc Psychiatry Psychiatr Epidemiol. Jul 2020;55(7):877-889. [doi: ] [Medline: 30874828]

28. Achtyes ED, Ben-Zeev D, Luo Z, et al. Off-hours use of a smartphone intervention to extend support for individuals with schizophrenia spectrum disorders recently discharged from a psychiatric hospital. Schizophr Res. Apr 2019;206:200-208. [doi: ] [Medline: 30551981]

29. Ben-Zeev D, Brenner CJ, Begale M, Duffecy J, Mohr DC, Mueser KT. Feasibility, acceptability, and preliminary efficacy of a smartphone intervention for schizophrenia. Schizophr Bull. Nov 2014;40(6):1244-1253. [doi: ] [Medline: 24609454]

30. Ben-Zeev D, Brian RM, Aschbrenner KA, Jonathan G, Steingard S. Video-based mobile health interventions for people with schizophrenia: Bringing the “pocket therapist” to life. Psychiatr Rehabil J. Mar 2018;41(1):39-45. [doi: ] [Medline: 27295133]

31. Sedgwick O, Hardy A, Greer B, Newbery K, Cella M. “I wanted to do more of the homework!”-Feasibility and acceptability of blending app-based homework with group therapy for social cognition in psychosis. J Clin Psychol. Dec 2021;77(12):2701-2724. [doi: ] [Medline: 34101177]

32. Austin SF, Jansen JE, Petersen CJ, Jensen R, Simonsen E. Mobile App Integration Into Dialectical Behavior Therapy for Persons With Borderline Personality Disorder: Qualitative and Quantitative Study. JMIR Ment Health. Jun 11, 2020;7(6):e14913. [doi: ] [Medline: 32525488]

33. von Malachowski A, Schlier B, Austin SF, et al. IMPACHS: Feasibility and acceptability of an m-health solution integrated into routine clinical treatment for psychosis. Schizophr Res. Feb 2022;240:150-152. [doi: ] [Medline: 35026600]

34. Fulford D, Gard DE, Mueser KT, et al. Preliminary Outcomes of an Ecological Momentary Intervention for Social Functioning in Schizophrenia: Pre-Post Study of the Motivation and Skills Support App. JMIR Ment Health. Jun 15, 2021;8(6):e27475. [doi: ] [Medline: 34128812]

35. Krzystanek M, Borkowski M, Skałacka K, Krysta K. A telemedicine platform to improve clinical parameters in paranoid schizophrenia patients: Results of a one-year randomized study. Schizophr Res. Feb 2019;204:389-396. [doi: ] [Medline: 30154027]

36. Krzystanek M, Krysta K, Borkowski M, et al. The Effect of Smartphone-Based Cognitive Training on the Functional/Cognitive Markers of Schizophrenia: A One-Year Randomized Study. J Clin Med. Nov 16, 2020;9(11):3681. [doi: ] [Medline: 33207811]

37. Steare T, O’Hanlon P, Eskinazi M, et al. Smartphone-delivered self-management for first-episode psychosis: the ARIES feasibility randomised controlled trial. BMJ Open. Aug 26, 2020;10(8):e034927. [doi: ] [Medline: 32847902]

38. Steare T, Giorgalli M, Free K, et al. A qualitative study of stakeholder views on the use of a digital app for supported self-management in early intervention services for psychosis. BMC Psychiatry. Jun 19, 2021;21(1):311. [doi: ] [Medline: 34147075]

39. Han M, Lee K, Kim M, Heo Y, Choi H. Effects of a Metacognitive Smartphone Intervention With Weekly Mentoring Sessions for Individuals With Schizophrenia: A Quasi-Experimental Study. J Psychosoc Nurs Ment Health Serv. Feb 2023;61(2):27-37. [doi: ] [Medline: 35858205]

40. Ghaemi SN, Sverdlov O, van Dam J, Campellone T, Gerwien R. A Smartphone-Based Intervention as an Adjunct to Standard-of-Care Treatment for Schizophrenia: Randomized Controlled Trial. JMIR Form Res. Mar 28, 2022;6(3):e29154. [doi: ] [Medline: 35343910]

41. Fisher M, Etter K, Murray A, et al. The Effects of Remote Cognitive Training Combined With a Mobile App Intervention on Psychosis: Double-Blind Randomized Controlled Trial. J Med Internet Res. Nov 13, 2023;25(1):e48634. [doi: ] [Medline: 37955951]

42. Bell IH, Rossell SL, Farhall J, et al. Pilot randomised controlled trial of a brief coping-focused intervention for hearing voices blended with smartphone-based ecological momentary assessment and intervention (SAVVy): Feasibility, acceptability and preliminary clinical outcomes. Schizophr Res. Feb 2020;216:479-487. [doi: ] [Medline: 31812327]

43. Bell IH, Fielding-Smith SF, Hayward M, et al. Smartphone-based ecological momentary assessment and intervention in a blended coping-focused therapy for distressing voices: Development and case illustration. Internet Interv. Dec 2018;14:18-25. [doi: ] [Medline: 30510910]

44. Taylor KM, Bradley J, Cella M. A novel smartphone-based intervention targeting sleep difficulties in individuals experiencing psychosis: A feasibility and acceptability evaluation. Psychol Psychother. Sep 2022;95(3):717-737. [doi: ] [Medline: 35481697]

45. Garety PA, Ward T, Freeman D, et al. SlowMo, a digital therapy targeting reasoning in paranoia, versus treatment as usual in the treatment of people who fear harm from others: study protocol for a randomised controlled trial. Trials. Nov 2, 2017;18(1):510. [doi: ] [Medline: 29096681]

46. Garety P, Ward T, Emsley R, et al. Digitally supported CBT to reduce paranoia and improve reasoning for people with schizophrenia-spectrum psychosis: the SlowMo RCT. Efficacy Mech Eval. 2021;8(11):1-90. [doi: ] [Medline: 34398537]

47. Hanssen E, Balvert S, Oorschot M, et al. An ecological momentary intervention incorporating personalised feedback to improve symptoms and social functioning in schizophrenia spectrum disorders. Psychiatry Res. Feb 2020;284:112695. [doi: ] [Medline: 31831201]

48. Husain N, Gire N, Kelly J, et al. TechCare: mobile assessment and therapy for psychosis - an intervention for clients in the Early Intervention Service: A feasibility study protocol. SAGE Open Med. 2016;4:2050312116669613. [doi: ] [Medline: 27790373]

49. Gire N, Caton N, McKeown M, et al. ’Care co-ordinator in my pocket’: a feasibility study of mobile assessment and therapy for psychosis (TechCare). BMJ Open. Nov 16, 2021;11(11):e046755. [doi: ] [Medline: 34785541]

50. Jongeneel A, Libedinsky I, Reinbergen A, et al. Momentary effects of Temstem, an app for voice-hearing individuals: Results from naturalistic data from 1048 users. Internet Interv. Dec 2022;30:100580. [doi: 10.1016/j.invent.2022.100580] [Medline: 36277314]

51. de Almeida RS, Couto A, Marques A, Queirós C, Martins C. Mobile Application for Self-Management in Schizophrenia: A Pilot Study. J Technol Hum Serv. Oct 2, 2018;36(4):179-190. [doi: ]
