# Supplementary material for: Persuasive Systems Design Features of Smartphone Apps for Psychosis: Systematic Review
Source: JMIR Hum Factors. 2026 May 7;13:e81101. doi: 10.2196/81101 (PMC13151958; doi:10.2196/81101)
Supplement: Multimedia Appendix 2 [file humanfactors-v13-e81101-s002.docx]

| *App name* | *Reduction* | *Tunnelling* | *Tailoring* | *Personalization* | *Self-*  *monitoring* | *Simulation* | *Rehearsal* | *Praise* | *Rewards* | *Reminders* | *Suggestion* | *Similarity* | *Liking* | *Social*  *role* | *Trustworthiness* | *Expertise* | *Real-world*  *feel* | *Social*  *learning* | *Social*  *comparison* | *Normative*  *influence* | *Social*  *facilitation* |
| --- | --- | --- | --- | --- | --- | --- | --- | --- | --- | --- | --- | --- | --- | --- | --- | --- | --- | --- | --- | --- | --- |
| A4i [22, 23] |  | x |  | x | x |  |  |  |  | x | x |  |  | x |  |  |  |  |  | x | x |
| Actissist [24] |  | x |  | x | x |  |  |  |  | x | x | x | x |  |  |  | x |  |  |  |  |
| Chilltime [25] | x |  |  | x |  |  |  |  |  | x | x |  | x |  |  | x | x |  |  |  |  |
| CBT2go [26] |  | x |  | x | x |  | x | x | x | x | x |  |  | x |  |  |  |  |  |  |  |
| Connect+ [27] | x | x |  |  | x | x |  |  | x | x | x | x | x |  | x |  |  | x |  |  |  |
| Focus [28, 29] |  |  |  | x |  |  |  |  |  |  | x |  | x |  |  |  |  |  |  |  |  |
| Focus-AV [30] | x |  |  | x |  | x | x |  |  |  |  |  |  |  |  |  | x |  |  |  |  |
| Grasp [31] | x | x |  |  |  |  | x |  |  | x |  |  |  |  |  |  |  |  |  |  |  |
| IMPACHS [32, 33] | x | x |  | x | x |  |  |  |  |  | x |  | x | x |  |  |  |  |  |  |  |
| MASS [34] | x | x | x | x | x | x |  | x |  | x | x | x |  |  |  |  |  | x |  |  |  |
| Moneo [35, 36] |  |  |  |  |  |  |  |  |  | x |  |  |  | x |  |  |  |  |  |  |  |
| My Journey [37, 38] |  |  |  | x | x |  |  |  |  | x |  |  | x | x |  |  |  |  |  |  |  |
| MCI-S [39] |  | x |  |  |  |  |  |  |  |  |  | x |  |  |  |  |  |  |  |  |  |
| PEAR004 [40] |  |  |  | x |  |  |  |  |  | x | x |  |  |  |  |  |  |  |  |  |  |
| PRIME [41] |  | x |  | x | x |  |  | x |  |  | x |  | x | x |  |  |  | x | x | x | x |
| Savvy [42, 43] |  |  |  | x | x |  | x |  |  | x |  |  |  |  |  |  |  |  |  |  |  |
| Sleep app [44] | x |  | x | x |  |  |  | x | x | x |  | x |  |  |  |  |  |  |  |  |  |
| SlowMo [45, 46] |  |  |  | x |  |  | x |  |  | x |  |  | x |  |  |  |  |  |  |  |  |
| SMART app [47] |  |  |  | x |  |  |  |  |  | x | x |  |  |  |  |  |  |  |  |  |  |
| TechCare [48, 49] |  |  |  | x |  |  |  |  |  | x |  |  |  |  |  |  |  |  |  |  |  |
| TemStem [50] | x | x |  | x | x |  |  | x | x |  |  |  |  |  |  |  |  |  |  |  |  |
| WeCOPE [51] | x |  |  | x |  |  |  |  |  |  |  |  |  |  |  |  |  |  |  |  |  |

References:

22. Kidd SA, Feldcamp L, Adler A, et al. Feasibility and outcomes of a multi-function mobile health approach for the schizophrenia spectrum: App4Independence (A4i). PLOS ONE. 2019;14(7):e0219491. [doi: ] [Medline: 31306439]

23. Kidd SA, D’Arcey J, Tackaberry-Giddens L, et al. App for independence: A feasibility randomized controlled trial of a digital health tool for schizophrenia spectrum disorders. Schizophr Res. Jan 2025;275:52-61. [doi: ] [Medline: 39657429]

24. Bucci S, Barrowclough C, Ainsworth J, et al. Actissist: Proof-of-Concept Trial of a Theory-Driven Digital Intervention for Psychosis. Schizophr Bull. Aug 20, 2018;44(5):1070-1080. [doi: ] [Medline: 29566206]

25. Pennou A, Lecomte T, Potvin S, et al. A Mobile Health App (ChillTime) Promoting Emotion Regulation in Dual Disorders: Acceptability and Feasibility Pilot Study. JMIR Form Res. Jan 27, 2023;7(1):e37293. [doi: ] [Medline: 36705963]

26. Granholm E, Holden J, Dwyer K, Mikhael T, Link P, Depp C. Mobile-Assisted Cognitive Behavioral Therapy for Negative Symptoms: Open Single-Arm Trial With Schizophrenia Patients. JMIR Ment Health. Dec 1, 2020;7(12):e24406. [doi: ] [Medline: 33258792]

27. Lim MH, Gleeson JFM, Rodebaugh TL, et al. A pilot digital intervention targeting loneliness in young people with psychosis. Soc Psychiatry Psychiatr Epidemiol. Jul 2020;55(7):877-889. [doi: ] [Medline: 30874828]

28. Achtyes ED, Ben-Zeev D, Luo Z, et al. Off-hours use of a smartphone intervention to extend support for individuals with schizophrenia spectrum disorders recently discharged from a psychiatric hospital. Schizophr Res. Apr 2019;206:200-208. [doi: ] [Medline: 30551981]

29. Ben-Zeev D, Brenner CJ, Begale M, Duffecy J, Mohr DC, Mueser KT. Feasibility, acceptability, and preliminary efficacy of a smartphone intervention for schizophrenia. Schizophr Bull. Nov 2014;40(6):1244-1253. [doi: ] [Medline: 24609454]

30. Ben-Zeev D, Brian RM, Aschbrenner KA, Jonathan G, Steingard S. Video-based mobile health interventions for people with schizophrenia: Bringing the “pocket therapist” to life. Psychiatr Rehabil J. Mar 2018;41(1):39-45. [doi: ] [Medline: 27295133]

31. Sedgwick O, Hardy A, Greer B, Newbery K, Cella M. “I wanted to do more of the homework!”-Feasibility and acceptability of blending app-based homework with group therapy for social cognition in psychosis. J Clin Psychol. Dec 2021;77(12):2701-2724. [doi: ] [Medline: 34101177]

32. Austin SF, Jansen JE, Petersen CJ, Jensen R, Simonsen E. Mobile App Integration Into Dialectical Behavior Therapy for Persons With Borderline Personality Disorder: Qualitative and Quantitative Study. JMIR Ment Health. Jun 11, 2020;7(6):e14913. [doi: ] [Medline: 32525488]

33. von Malachowski A, Schlier B, Austin SF, et al. IMPACHS: Feasibility and acceptability of an m-health solution integrated into routine clinical treatment for psychosis. Schizophr Res. Feb 2022;240:150-152. [doi: ] [Medline: 35026600]

34. Fulford D, Gard DE, Mueser KT, et al. Preliminary Outcomes of an Ecological Momentary Intervention for Social Functioning in Schizophrenia: Pre-Post Study of the Motivation and Skills Support App. JMIR Ment Health. Jun 15, 2021;8(6):e27475. [doi: ] [Medline: 34128812]

35. Krzystanek M, Borkowski M, Skałacka K, Krysta K. A telemedicine platform to improve clinical parameters in paranoid schizophrenia patients: Results of a one-year randomized study. Schizophr Res. Feb 2019;204:389-396. [doi: ] [Medline: 30154027]

36. Krzystanek M, Krysta K, Borkowski M, et al. The Effect of Smartphone-Based Cognitive Training on the Functional/Cognitive Markers of Schizophrenia: A One-Year Randomized Study. J Clin Med. Nov 16, 2020;9(11):3681. [doi: ] [Medline: 33207811]

37. Steare T, O’Hanlon P, Eskinazi M, et al. Smartphone-delivered self-management for first-episode psychosis: the ARIES feasibility randomised controlled trial. BMJ Open. Aug 26, 2020;10(8):e034927. [doi: ] [Medline: 32847902]

38. Steare T, Giorgalli M, Free K, et al. A qualitative study of stakeholder views on the use of a digital app for supported self-management in early intervention services for psychosis. BMC Psychiatry. Jun 19, 2021;21(1):311. [doi: ] [Medline: 34147075]

39. Han M, Lee K, Kim M, Heo Y, Choi H. Effects of a Metacognitive Smartphone Intervention With Weekly Mentoring Sessions for Individuals With Schizophrenia: A Quasi-Experimental Study. J Psychosoc Nurs Ment Health Serv. Feb 2023;61(2):27-37. [doi: ] [Medline: 35858205]

40. Ghaemi SN, Sverdlov O, van Dam J, Campellone T, Gerwien R. A Smartphone-Based Intervention as an Adjunct to Standard-of-Care Treatment for Schizophrenia: Randomized Controlled Trial. JMIR Form Res. Mar 28, 2022;6(3):e29154. [doi: ] [Medline: 35343910]

41. Fisher M, Etter K, Murray A, et al. The Effects of Remote Cognitive Training Combined With a Mobile App Intervention on Psychosis: Double-Blind Randomized Controlled Trial. J Med Internet Res. Nov 13, 2023;25(1):e48634. [doi: ] [Medline: 37955951]

42. Bell IH, Rossell SL, Farhall J, et al. Pilot randomised controlled trial of a brief coping-focused intervention for hearing voices blended with smartphone-based ecological momentary assessment and intervention (SAVVy): Feasibility, acceptability and preliminary clinical outcomes. Schizophr Res. Feb 2020;216:479-487. [doi: ] [Medline: 31812327]

43. Bell IH, Fielding-Smith SF, Hayward M, et al. Smartphone-based ecological momentary assessment and intervention in a blended coping-focused therapy for distressing voices: Development and case illustration. Internet Interv. Dec 2018;14:18-25. [doi: ] [Medline: 30510910]

44. Taylor KM, Bradley J, Cella M. A novel smartphone-based intervention targeting sleep difficulties in individuals experiencing psychosis: A feasibility and acceptability evaluation. Psychol Psychother. Sep 2022;95(3):717-737. [doi: ] [Medline: 35481697]

45. Garety PA, Ward T, Freeman D, et al. SlowMo, a digital therapy targeting reasoning in paranoia, versus treatment as usual in the treatment of people who fear harm from others: study protocol for a randomised controlled trial. Trials. Nov 2, 2017;18(1):510. [doi: ] [Medline: 29096681]

46. Garety P, Ward T, Emsley R, et al. Digitally supported CBT to reduce paranoia and improve reasoning for people with schizophrenia-spectrum psychosis: the SlowMo RCT. Efficacy Mech Eval. 2021;8(11):1-90. [doi: ] [Medline: 34398537]

47. Hanssen E, Balvert S, Oorschot M, et al. An ecological momentary intervention incorporating personalised feedback to improve symptoms and social functioning in schizophrenia spectrum disorders. Psychiatry Res. Feb 2020;284:112695. [doi: ] [Medline: 31831201]

48. Husain N, Gire N, Kelly J, et al. TechCare: mobile assessment and therapy for psychosis - an intervention for clients in the Early Intervention Service: A feasibility study protocol. SAGE Open Med. 2016;4:2050312116669613. [doi: ] [Medline: 27790373]

49. Gire N, Caton N, McKeown M, et al. ’Care co-ordinator in my pocket’: a feasibility study of mobile assessment and therapy for psychosis (TechCare). BMJ Open. Nov 16, 2021;11(11):e046755. [doi: ] [Medline: 34785541]

50. Jongeneel A, Libedinsky I, Reinbergen A, et al. Momentary effects of Temstem, an app for voice-hearing individuals: Results from naturalistic data from 1048 users. Internet Interv. Dec 2022;30:100580. [doi: 10.1016/j.invent.2022.100580] [Medline: 36277314]

51. de Almeida RS, Couto A, Marques A, Queirós C, Martins C. Mobile Application for Self-Management in Schizophrenia: A Pilot Study. J Technol Hum Serv. Oct 2, 2018;36(4):179-190. [doi: ]
